# 3-(Trimethyl­sil­yl)prop-2-ynyl *p*-toluene­sulfonate

**DOI:** 10.1107/S1600536811043595

**Published:** 2011-10-29

**Authors:** Devin C. Schmitt, Guillermo A. Morales, Frank R. Fronczek, Steven F. Watkins

**Affiliations:** a Department of Chemistry, Louisiana State University, Baton Rouge, LA 70803-1804, USA

## Abstract

In the title compound, C_13_H_18_O_3_SSi, the SO_3_ group displays a partial rotational (*ca* 50°) disorder about the C—S bond, with relative proportions 0.7744 (13):0.2256 (13). This disorder also forces the propynyl CH_2_ group to be disordered.

## Related literature

For information on the title compound, see: Westmijze & Vermeer (1979 ▶); Tanabe *et al.* (1995 ▶); Morales (1995 ▶).
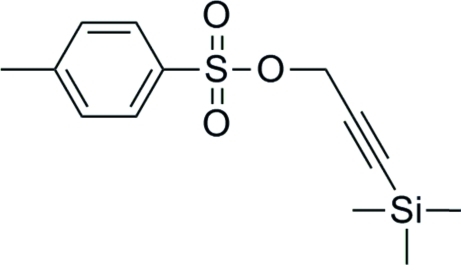


## Experimental

### 

#### Crystal data

C_13_H_18_O_3_SSi
*M* *_r_* = 282.42
Monoclinic, 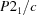

*a* = 10.6857 (4) Å
*b* = 12.9413 (5) Å
*c* = 11.8793 (4) Å
β = 113.471 (2)°
*V* = 1506.83 (10) Å^3^
*Z* = 4
Mo *K*α radiation
μ = 0.29 mm^−1^
*T* = 90 K
0.33 × 0.18 × 0.17 mm

#### Data collection

Nonius KappaCCD diffractometer
Absorption correction: multi-scan (*SCALEPACK*; Otwinowski & Minor, 1997 ▶) *T*_min_ = 0.910, *T*_max_ = 0.952
9135 measured reflections
4721 independent reflections
3257 reflections with *I* > 2σ(*I*)
*R*_int_ = 0.041

#### Refinement

*R*[*F* ^2^ > 2σ(*F* ^2^)] = 0.045
*wR*(*F* ^2^) = 0.108
*S* = 1.03
4721 reflections
177 parameters
1 restraint
H-atom parameters constrained
Δρ_max_ = 0.76 e Å^−3^
Δρ_min_ = −0.54 e Å^−3^

### 

Data collection: *COLLECT* (Nonius, 2000 ▶); cell refinement: *DENZO*/*SCALEPACK* (Otwinowski & Minor, 1997 ▶); data reduction: *DENZO*/*SCALEPACK*; program(s) used to solve structure: *SHELXS97* (Sheldrick, 2008 ▶); program(s) used to refine structure: *SHELXL97* (Sheldrick, 2008 ▶); molecular graphics: *ORTEP-3 for Windows* (Farrugia, 1997 ▶); software used to prepare material for publication: *WinGX* (Farrugia, 1999 ▶).

## Supplementary Material

Crystal structure: contains datablock(s) global, I. DOI: 10.1107/S1600536811043595/fj2461sup1.cif

Structure factors: contains datablock(s) I. DOI: 10.1107/S1600536811043595/fj2461Isup2.hkl

Supplementary material file. DOI: 10.1107/S1600536811043595/fj2461Isup3.cml

Additional supplementary materials:  crystallographic information; 3D view; checkCIF report

## Figures and Tables

**Fig. 1.**
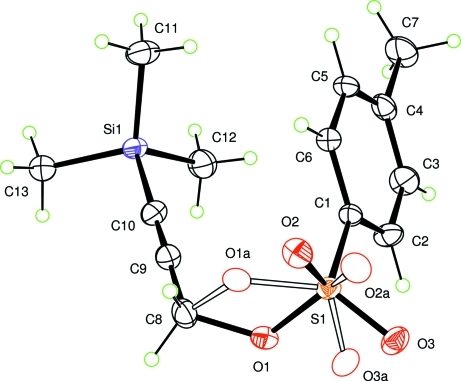
View of (I) showing 50% probability displacement ellipsoids. The two disordered components of the sulfonate group are shown, while only the major component of the attached disordered methylene group is drawn.

## supplementary crystallographic information

### Comment

The title compound was prepared as an intermediate in the synthesis of 3-methyl substituted benz[*f*]indole derivatives (Morales, 1995). It has been described as a colorless liquid at room temperature (Tanabe *et al.*, 1995; Westmijze & Vermeer, 1979). Our initial synthesis, by treating 3-trimethylsilylpropargyl alcohol with freshly powdered KOH and freshly recrystallized *p*-toluenesulfonyl chloride in ether at -50°C, also yielded the desired sulfonate as a liquid. However, after several recrystallizations from hexanes, suitable single crystals were obtained as colorless needles with melting point 43–44°C.

3-Trimethylsilyl-2-propynyl *p*-toluenesulfonate contains a *p*-toluenesulfonate group with the S(O)_2_OCH_2_ group partially (22.56 (13)%) disordered by rotation of *ca* 50° about the C–S bond. The disorder was modelled with constrained partial occupancy of two O_3_ and two H_2_ sites. The disordered O–C bond distances were restrained to be approximately equal (to within σ = 0.002 Å) to compensate for disorder of the C atom itself. The two partial S–O bonds average 1.574 (2) Å, and the four partial S═O bonds average 1.435 (1) Å.

### Experimental

The title compound was prepared according to the procedure of Westmijze & Vermeer (1979): Commercially available 3-trimethylsilylpropargyl alcohol (5.13 g, 40.0 mmol) and freshly purified tosyl chloride (9.53 g, 50.0 mmol) were mixed in anhydrous diethyl ether under an Ar atmosphere. The mixture was cooled to -50 °C, and freshly powdered KOH (15.0 g, 268 mmol) was added at once under vigorous stirring. The temperature of the resulting thick mixture was slowly raised to 0 °C, and the mixture was stirred at this temperature for 30 min. This mixture was poured onto water (200 ml), extracted with ethyl ether (3 τimes 50 ml), washed with water (100 ml), and concentrated under reduced pressure to give a light brown oil. Repeated crystallization from hexanes afforded colorless needles (7.93 g, 70.3% yield).

### Refinement

The site occupation factor was constrained to be *x* for O1, O2, O3, H8A, H8B, and 1 - *x* for O1A, O2A, O3A, H8A', H8B'. Atomic displacement parameters were constrained to be equal for the following disordered atom pairs: O1 and O1A, O2 and O2A, O3 and O3A, and interatomic distances C8—O1 and C8—O1A were restrained to be equal to within σ = 0.002.

All H atoms were placed in calculated positions, guided by difference maps, with C—H bond distances 0.95 (aromatic-H), 0.98 (methyl-H), and 0.99 (alkyl-H) Å, and displacement parameters *U*_iso_=1.2*U*_eq_ (aromatic and alkyl C) and 1.5*U*_eq_ (methyl-C), and thereafter refined as riding.

### Crystal data

**Table:**

C_13_H_18_O_3_SSi	*F*(000) = 600
*M**_r_* = 282.42	*D*_x_ = 1.245 Mg m^−^^3^
Monoclinic, *P*2_1_/*c*	Mo *K*α radiation, λ = 0.71073 Å
Hall symbol: -P 2ybc	Cell parameters from 4768 reflections
*a* = 10.6857 (4) Å	θ = 2.6–31.0°
*b* = 12.9413 (5) Å	µ = 0.29 mm^−^^1^
*c* = 11.8793 (4) Å	*T* = 90 K
β = 113.471 (2)°	Needle, colorless
*V* = 1506.83 (10) Å^3^	0.33 × 0.18 × 0.17 mm
*Z* = 4	

### Data collection

**Table:**

Nonius KappaCCD diffractometer	4721 independent reflections
Radiation source: fine-focus sealed tube	3257 reflections with *I* > 2σ(*I*)
graphite	*R*_int_ = 0.041
Detector resolution: 9 pixels mm^-1^	θ_max_ = 30.9°, θ_min_ = 2.6°
ω and φ scans	*h* = −15→15
Absorption correction: multi-scan (*SCALEPACK*; Otwinowski & Minor, 1997)	*k* = −18→18
*T*_min_ = 0.910, *T*_max_ = 0.952	*l* = −17→16
9135 measured reflections	

### Refinement

**Table:**

Refinement on *F*^2^	Primary atom site location: structure-invariant direct methods
Least-squares matrix: full	Secondary atom site location: difference Fourier map
*R*[*F*^2^ > 2σ(*F*^2^)] = 0.045	Hydrogen site location: inferred from neighbouring sites
*wR*(*F*^2^) = 0.108	H-atom parameters constrained
*S* = 1.03	*w* = 1/[σ^2^(*F*_o_^2^) + (0.0404*P*)^2^ + 0.7293*P*] where *P* = (*F*_o_^2^ + 2*F*_c_^2^)/3
4721 reflections	(Δ/σ)_max_ = 0.001
177 parameters	Δρ_max_ = 0.76 e Å^−^^3^
1 restraint	Δρ_min_ = −0.54 e Å^−^^3^
3 constraints	

### Special details

**Table:**

Geometry. All e.s.d.'s (except the e.s.d. in the dihedral angle between two l.s. planes) are estimated using the full covariance matrix. The cell e.s.d.'s are taken into account individually in the estimation of e.s.d.'s in distances, angles and torsion angles; correlations between e.s.d.'s in cell parameters are only used when they are defined by crystal symmetry. An approximate (isotropic) treatment of cell e.s.d.'s is used for estimating e.s.d.'s involving l.s. planes.

### Fractional atomic coordinates and isotropic or equivalent isotropic displacement parameters (Å^2^)

**Table:**

	*x*	*y*	*z*	*U*_iso_*/*U*_eq_	Occ. (<1)
S1	1.00481 (4)	0.33704 (3)	0.46346 (4)	0.01708 (10)	
Si1	0.49810 (5)	0.23584 (4)	0.04270 (4)	0.02127 (12)	
O1	0.87983 (15)	0.41268 (11)	0.42804 (13)	0.0207 (3)	0.7744 (13)
O2	1.08780 (16)	0.36473 (12)	0.39833 (15)	0.0237 (3)	0.7744 (13)
O3	1.06468 (17)	0.33877 (12)	0.59590 (14)	0.0250 (3)	0.7744 (13)
O1A	0.9453 (3)	0.4058 (4)	0.3433 (4)	0.0207 (3)	0.2256 (13)
O2A	1.1404 (6)	0.3363 (4)	0.4827 (5)	0.0237 (3)	0.2256 (13)
O3A	0.9569 (6)	0.3761 (4)	0.5540 (5)	0.0250 (3)	0.2256 (13)
C1	0.93291 (17)	0.21448 (12)	0.41521 (15)	0.0178 (3)	
C2	0.86434 (19)	0.16667 (14)	0.47865 (16)	0.0240 (4)	
H2	0.8564	0.1997	0.5469	0.029*	
C3	0.8077 (2)	0.06981 (14)	0.44071 (17)	0.0282 (4)	
H3	0.7606	0.0364	0.4836	0.034*	
C4	0.81874 (18)	0.02064 (13)	0.34052 (16)	0.0257 (4)	
C5	0.88776 (18)	0.07073 (13)	0.27880 (16)	0.0246 (4)	
H5	0.8955	0.038	0.2102	0.03*	
C6	0.94568 (17)	0.16762 (13)	0.31531 (15)	0.0212 (3)	
H6	0.993	0.2011	0.2728	0.025*	
C7	0.7575 (2)	−0.08509 (15)	0.3004 (2)	0.0377 (5)	
H7A	0.7553	−0.1005	0.2188	0.057*	
H7C	0.6645	−0.0864	0.2972	0.057*	
H7B	0.8131	−0.137	0.3591	0.057*	
C8	0.81275 (19)	0.45147 (14)	0.30416 (16)	0.0272 (4)	
H8A	0.8829	0.4663	0.2715	0.033*	0.7744 (13)
H8B	0.7671	0.5175	0.3066	0.033*	0.7744 (13)
H8A'	0.8112	0.518	0.2626	0.033*	0.2256 (13)
H8B'	0.7906	0.4652	0.3762	0.033*	0.2256 (13)
C9	0.71152 (19)	0.38092 (14)	0.21960 (16)	0.0237 (4)	
C10	0.62740 (19)	0.32454 (14)	0.14905 (16)	0.0247 (4)	
C11	0.58308 (19)	0.15508 (16)	−0.03569 (18)	0.0314 (4)	
H11A	0.6075	0.1983	−0.0916	0.047*	
H11B	0.5207	0.1003	−0.0824	0.047*	
H11C	0.6658	0.1241	0.0257	0.047*	
C12	0.4334 (2)	0.15281 (15)	0.13563 (19)	0.0333 (4)	
H12A	0.51	0.1164	0.1983	0.05*	
H12B	0.3686	0.1023	0.0821	0.05*	
H12C	0.3876	0.1959	0.1755	0.05*	
C13	0.36123 (19)	0.31849 (14)	−0.06735 (17)	0.0270 (4)	
H13A	0.3137	0.355	−0.0237	0.041*	
H13B	0.2962	0.2751	−0.1317	0.041*	
H13C	0.4018	0.3688	−0.1045	0.041*	

### Atomic displacement parameters (Å^2^)

**Table:**

	*U*^11^	*U*^22^	*U*^33^	*U*^12^	*U*^13^	*U*^23^
S1	0.01972 (19)	0.01537 (18)	0.01643 (19)	−0.00309 (16)	0.00751 (15)	−0.00186 (15)
Si1	0.0256 (2)	0.0199 (2)	0.0190 (2)	−0.0004 (2)	0.00962 (19)	−0.00338 (19)
O1	0.0220 (7)	0.0174 (7)	0.0228 (8)	−0.0009 (6)	0.0091 (6)	−0.0038 (6)
O2	0.0242 (8)	0.0217 (8)	0.0295 (8)	−0.0020 (6)	0.0152 (7)	0.0019 (6)
O3	0.0322 (8)	0.0222 (8)	0.0168 (7)	−0.0078 (7)	0.0058 (6)	−0.0022 (6)
O1A	0.0220 (7)	0.0174 (7)	0.0228 (8)	−0.0009 (6)	0.0091 (6)	−0.0038 (6)
O2A	0.0242 (8)	0.0217 (8)	0.0295 (8)	−0.0020 (6)	0.0152 (7)	0.0019 (6)
O3A	0.0322 (8)	0.0222 (8)	0.0168 (7)	−0.0078 (7)	0.0058 (6)	−0.0022 (6)
C1	0.0199 (8)	0.0136 (7)	0.0176 (8)	−0.0008 (6)	0.0050 (6)	−0.0020 (6)
C2	0.0311 (9)	0.0206 (8)	0.0227 (8)	−0.0059 (7)	0.0134 (7)	−0.0051 (7)
C3	0.0346 (10)	0.0207 (9)	0.0307 (10)	−0.0075 (8)	0.0144 (8)	−0.0006 (7)
C4	0.0253 (9)	0.0159 (8)	0.0268 (9)	0.0007 (7)	0.0006 (7)	−0.0028 (7)
C5	0.0294 (9)	0.0199 (8)	0.0200 (8)	0.0056 (7)	0.0049 (7)	−0.0058 (7)
C6	0.0246 (8)	0.0203 (8)	0.0184 (8)	0.0035 (7)	0.0083 (7)	−0.0008 (7)
C7	0.0426 (12)	0.0185 (9)	0.0417 (12)	−0.0053 (8)	0.0059 (10)	−0.0078 (8)
C8	0.0314 (9)	0.0178 (8)	0.0256 (9)	0.0002 (7)	0.0043 (8)	0.0018 (7)
C9	0.0291 (9)	0.0212 (8)	0.0224 (9)	0.0032 (7)	0.0120 (7)	0.0018 (7)
C10	0.0317 (10)	0.0241 (9)	0.0199 (8)	0.0008 (7)	0.0120 (7)	−0.0004 (7)
C11	0.0257 (9)	0.0394 (11)	0.0267 (9)	0.0020 (8)	0.0079 (8)	−0.0119 (8)
C12	0.0434 (12)	0.0255 (10)	0.0342 (11)	−0.0013 (9)	0.0191 (9)	0.0022 (8)
C13	0.0299 (9)	0.0251 (9)	0.0250 (9)	0.0012 (7)	0.0100 (8)	−0.0014 (7)

### Geometric parameters (Å, °)

**Table:**

S1—O2A	1.374 (5)	C5—C6	1.389 (2)
S1—O2	1.4352 (15)	C5—H5	0.95
S1—O3	1.4434 (15)	C6—H6	0.95
S1—O3A	1.454 (6)	C7—H7A	0.98
S1—O1	1.5720 (15)	C7—H7C	0.98
S1—O1A	1.584 (5)	C7—H7B	0.98
S1—C1	1.7561 (16)	C8—C9	1.465 (2)
Si1—C10	1.8526 (19)	C8—H8A	0.99
Si1—C11	1.8594 (19)	C8—H8B	0.99
Si1—C12	1.8600 (19)	C8—H8A'	0.99
Si1—C13	1.8625 (19)	C8—H8B'	0.99
O1—C8	1.446 (2)	C9—C10	1.201 (3)
O1A—C8	1.430 (3)	C11—H11A	0.98
C1—C6	1.387 (2)	C11—H11B	0.98
C1—C2	1.388 (2)	C11—H11C	0.98
C2—C3	1.387 (2)	C12—H12A	0.98
C2—H2	0.95	C12—H12B	0.98
C3—C4	1.396 (3)	C12—H12C	0.98
C3—H3	0.95	C13—H13A	0.98
C4—C5	1.390 (3)	C13—H13B	0.98
C4—C7	1.510 (2)	C13—H13C	0.98
			
O2—S1—O3	118.88 (10)	C4—C7—H7A	109.5
O2A—S1—O3A	122.5 (3)	C4—C7—H7C	109.5
O2—S1—O1	109.98 (9)	H7A—C7—H7C	109.5
O3—S1—O1	104.04 (9)	C4—C7—H7B	109.5
O2A—S1—O1A	99.9 (3)	H7A—C7—H7B	109.5
O3A—S1—O1A	109.7 (3)	H7C—C7—H7B	109.5
O2A—S1—C1	110.1 (2)	O1A—C8—C9	109.3 (2)
O2—S1—C1	109.48 (9)	O1—C8—C9	114.37 (15)
O3—S1—C1	108.43 (8)	O1—C8—H8A	108.7
O3A—S1—C1	108.6 (2)	C9—C8—H8A	108.7
O1—S1—C1	105.10 (8)	O1—C8—H8B	108.7
O1A—S1—C1	104.31 (19)	C9—C8—H8B	108.7
C10—Si1—C11	108.03 (9)	H8A—C8—H8B	107.6
C10—Si1—C12	107.80 (9)	O1A—C8—H8A'	109.8
C11—Si1—C12	110.10 (10)	C9—C8—H8A'	109.8
C10—Si1—C13	106.66 (8)	O1A—C8—H8B'	109.8
C11—Si1—C13	112.01 (9)	C9—C8—H8B'	109.8
C12—Si1—C13	112.01 (9)	H8A'—C8—H8B'	108.3
C8—O1—S1	120.83 (12)	C10—C9—C8	178.9 (2)
C8—O1A—S1	121.0 (3)	C9—C10—Si1	178.90 (17)
C6—C1—C2	121.51 (15)	Si1—C11—H11A	109.5
C6—C1—S1	119.66 (13)	Si1—C11—H11B	109.5
C2—C1—S1	118.83 (12)	H11A—C11—H11B	109.5
C3—C2—C1	118.87 (16)	Si1—C11—H11C	109.5
C3—C2—H2	120.6	H11A—C11—H11C	109.5
C1—C2—H2	120.6	H11B—C11—H11C	109.5
C2—C3—C4	121.00 (17)	Si1—C12—H12A	109.5
C2—C3—H3	119.5	Si1—C12—H12B	109.5
C4—C3—H3	119.5	H12A—C12—H12B	109.5
C5—C4—C3	118.69 (16)	Si1—C12—H12C	109.5
C5—C4—C7	120.67 (17)	H12A—C12—H12C	109.5
C3—C4—C7	120.64 (18)	H12B—C12—H12C	109.5
C6—C5—C4	121.35 (16)	Si1—C13—H13A	109.5
C6—C5—H5	119.3	Si1—C13—H13B	109.5
C4—C5—H5	119.3	H13A—C13—H13B	109.5
C1—C6—C5	118.58 (16)	Si1—C13—H13C	109.5
C1—C6—H6	120.7	H13A—C13—H13C	109.5
C5—C6—H6	120.7	H13B—C13—H13C	109.5
			
O2—S1—O1—C8	36.57 (16)	O3—S1—C1—C2	40.01 (17)
O3—S1—O1—C8	164.93 (14)	O3A—S1—C1—C2	−10.1 (3)
C1—S1—O1—C8	−81.18 (15)	O1—S1—C1—C2	−70.77 (15)
O2A—S1—O1A—C8	−163.6 (4)	O1A—S1—C1—C2	−127.00 (18)
O3A—S1—O1A—C8	−33.6 (5)	C6—C1—C2—C3	0.0 (3)
C1—S1—O1A—C8	82.5 (4)	S1—C1—C2—C3	−179.91 (14)
O2A—S1—C1—C6	−53.4 (3)	C1—C2—C3—C4	−0.1 (3)
O2—S1—C1—C6	−8.82 (17)	C2—C3—C4—C5	−0.1 (3)
O3—S1—C1—C6	−139.95 (14)	C2—C3—C4—C7	179.61 (18)
O3A—S1—C1—C6	170.0 (3)	C3—C4—C5—C6	0.3 (3)
O1—S1—C1—C6	109.28 (14)	C7—C4—C5—C6	−179.42 (17)
O1A—S1—C1—C6	53.05 (19)	C2—C1—C6—C5	0.1 (3)
O2A—S1—C1—C2	126.6 (3)	S1—C1—C6—C5	−179.91 (13)
O2—S1—C1—C2	171.14 (14)	C4—C5—C6—C1	−0.3 (3)
